# Supplementary material for: Risk factors for recovery from oculomotor nerve palsy after aneurysm surgery: a meta-analysis
Source: PeerJ. 2024 Oct 29;12:e18207. doi: 10.7717/peerj.18207 (PMC11529594; doi:10.7717/peerj.18207)
Supplement: Supplemental Information 2 [file peerj-12-18207-s002.docx]

((("Intracranial Aneurysm"[Mesh]) OR ((((((((((((((((((((((((((((((((((((((((((((Intracranial Aneurysm[Title/Abstract]) OR (Aneurysms, Intracranial[Title/Abstract])) OR (Intracranial Aneurysms[Title/Abstract])) OR (Aneurysm, Intracranial[Title/Abstract])) OR (Aneurysm, Anterior Communicating Artery[Title/Abstract])) OR (Anterior Communicating Artery Aneurysm[Title/Abstract])) OR (Aneurysm, Basilar Artery[Title/Abstract])) OR (Aneurysms, Basilar Artery[Title/Abstract])) OR (Artery Aneurysm, Basilar[Title/Abstract])) OR (Artery Aneurysms, Basilar[Title/Abstract])) OR (Basilar Artery Aneurysms[Title/Abstract])) OR (Basilar Artery Aneurysm[Title/Abstract])) OR (Aneurysm, Middle Cerebral Artery[Title/Abstract])) OR (Middle Cerebral Artery Aneurysm[Title/Abstract])) OR (Aneurysm, Posterior Cerebral Artery[Title/Abstract])) OR (Posterior Cerebral Artery Aneurysm[Title/Abstract])) OR (Berry Aneurysm[Title/Abstract])) OR (Aneurysm, Berry[Title/Abstract])) OR (Aneurysms, Berry[Title/Abstract])) OR (Berry Aneurysms[Title/Abstract])) OR (Brain Aneurysm[Title/Abstract])) OR (Aneurysm, Brain[Title/Abstract])) OR (Aneurysms, Brain[Title/Abstract])) OR (Brain Aneurysms[Title/Abstract])) OR (Cerebral Aneurysm[Title/Abstract])) OR (Aneurysms, Cerebral[Title/Abstract])) OR (Cerebral Aneurysms[Title/Abstract])) OR (Aneurysm, Cerebral[Title/Abstract])) OR (Giant Intracranial Aneurysm[Title/Abstract])) OR (Aneurysm, Giant Intracranial[Title/Abstract])) OR (Aneurysms, Giant Intracranial[Title/Abstract])) OR (Giant Intracranial Aneurysms[Title/Abstract])) OR (Intracranial Aneurysm, Giant[Title/Abstract])) OR (Intracranial Aneurysms, Giant[Title/Abstract])) OR (Mycotic Aneurysm, Intracranial[Title/Abstract])) OR (Aneurysm, Intracranial Mycotic[Title/Abstract])) OR (Aneurysms, Intracranial Mycotic[Title/Abstract])) OR (Intracranial Mycotic Aneurysm[Title/Abstract])) OR (Intracranial Mycotic Aneurysms[Title/Abstract])) OR (Mycotic Aneurysms, Intracranial[Title/Abstract])) OR (Aneurysm, Anterior Cerebral Artery[Title/Abstract])) OR (Anterior Cerebral Artery Aneurysm[Title/Abstract])) OR (Aneurysm, Posterior Communicating Artery[Title/Abstract])) OR (Posterior Communicating Artery Aneurysm[Title/Abstract]))) AND (("Oculomotor Nerve Diseases"[Mesh]) OR ((((((((((((((((((((((((((((((((((((((Oculomotor Nerve Diseases[Title/Abstract]) OR (Nerve Disease, Oculomotor[Title/Abstract])) OR (Oculomotor Nerve Disease[Title/Abstract])) OR (Third Cranial Nerve Diseases[Title/Abstract])) OR (Oculomotor Nerve Paralysis[Title/Abstract])) OR (Nerve Paralysis, Oculomotor[Title/Abstract])) OR (Oculomotor Nerve Paralyses[Title/Abstract])) OR (Paralysis, Oculomotor Nerve[Title/Abstract])) OR (Oculomotor Neuropathy[Title/Abstract])) OR (Neuropathy, Oculomotor[Title/Abstract])) OR (Oculomotor Neuropathies[Title/Abstract])) OR (Third-Nerve Paralysis[Title/Abstract])) OR (Paralysis, Third-Nerve[Title/Abstract])) OR (Third Nerve Paralysis[Title/Abstract])) OR (Third-Nerve Paralyses[Title/Abstract])) OR (Cranial Nerve III Diseases[Title/Abstract])) OR (Oculomotor Nerve Disorders[Title/Abstract])) OR (Nerve Disorder, Oculomotor[Title/Abstract])) OR (Oculomotor Nerve Disorder[Title/Abstract])) OR (Oculomotor Nerve Palsy[Title/Abstract])) OR (Nerve Palsy, Oculomotor[Title/Abstract])) OR (Oculomotor Nerve Palsies[Title/Abstract])) OR (Palsy, Oculomotor Nerve[Title/Abstract])) OR (Third-Nerve Palsy[Title/Abstract])) OR (Palsy, Third-Nerve[Title/Abstract])) OR (Third Nerve Palsy[Title/Abstract])) OR (Third-Nerve Palsies[Title/Abstract])) OR (Total Third-Nerve Palsy[Title/Abstract])) OR (Palsy, Total Third-Nerve[Title/Abstract])) OR (Third-Nerve Palsy, Total[Title/Abstract])) OR (Total Third Nerve Palsy[Title/Abstract])) OR (Total Third-Nerve Palsies[Title/Abstract])) OR (Partial Third-Nerve Palsy[Title/Abstract])) OR (Palsy, Partial Third-Nerve[Title/Abstract])) OR (Partial Third Nerve Palsy[Title/Abstract])) OR (Partial Third-Nerve Palsies[Title/Abstract])) OR (Third-Nerve Palsies, Partial[Title/Abstract])) OR (Third-Nerve Palsy, Partial[Title/Abstract])))) AND (("Risk Factors"[Mesh]) OR (((((((((((((((((((((Risk Factors[Title/Abstract]) OR (Factor, Risk[Title/Abstract])) OR (Risk Factor[Title/Abstract])) OR (Social Risk Factors[Title/Abstract])) OR (Factor, Social Risk[Title/Abstract])) OR (Factors, Social Risk[Title/Abstract])) OR (Risk Factor, Social[Title/Abstract])) OR (Risk Factors, Social[Title/Abstract])) OR (Social Risk Factor[Title/Abstract])) OR (Health Correlates[Title/Abstract])) OR (Correlates, Health[Title/Abstract])) OR (Population at Risk[Title/Abstract])) OR (Populations at Risk[Title/Abstract])) OR (Risk Scores[Title/Abstract])) OR (Risk Score[Title/Abstract])) OR (Score, Risk[Title/Abstract])) OR (Risk Factor Scores[Title/Abstract])) OR (Risk Factor Score[Title/Abstract])) OR (Score, Risk Factor[Title/Abstract])) OR (Influencing factors[Title/Abstract])) OR (Predictive factors[Title/Abstract])))
