# Supplementary material for: Risk factors for recovery from oculomotor nerve palsy after aneurysm surgery: a meta-analysis
Source: PeerJ. 2024 Oct 29;12:e18207. doi: 10.7717/peerj.18207 (PMC11529594; doi:10.7717/peerj.18207)
Supplement: Supplemental Information 3 [file peerj-12-18207-s003.docx]

Why did you do this study?

The present study is the first to use meta-analysis to assess the risk factors for poor recovery from oculomotor nerve palsy after aneurysm surgery, and it found that preoperative complete oculomotor nerve palsy, surgery, and subarachnoid hemorrhage were all risk factors for poor recovery after aneurysm surgery.

What does this study add to other studies?

gender (OR=0.75, 95%CI 0.51-1.10), age (OR=1.00, 95%CI 0.93-1.07), aneurysm size (OR=0.85, 95%CI -0.71-1.01), treatment time (OR=1.01, 95%CI 0.91-1.13) is not a risk factor for recovery of motor nerve palsy after aneurysm surgery. Preoperative complete ONP (OR=2.27, 95%CI 1.07-4.81), surgery (OR=9.88, 95%CI 2.53-38.57), subarachnoid hemorrhage (OR=1.29, 95%CI 1.06-1.56) is a risk factor for recovery of motor nerve palsy after aneurysm surgery.
