# Supplementary material for: Risk factors for recovery from oculomotor nerve palsy after aneurysm surgery: a meta-analysis
Source: PeerJ. 2024 Oct 29;12:e18207. doi: 10.7717/peerj.18207 (PMC11529594; doi:10.7717/peerj.18207)
Supplement: Supplemental Information 4 [file peerj-12-18207-s004.docx]

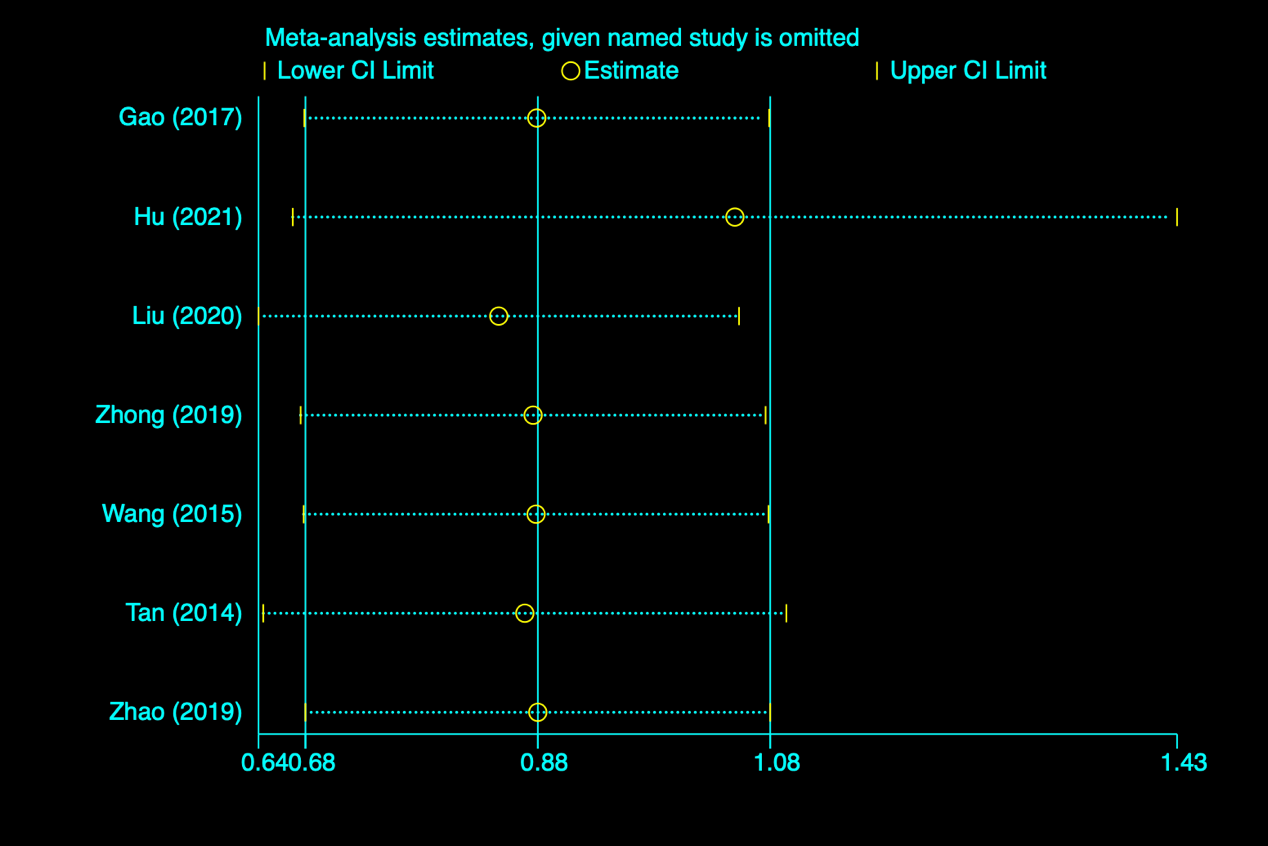


figure s1 Sensitivity analysis of preoperative complete ONP


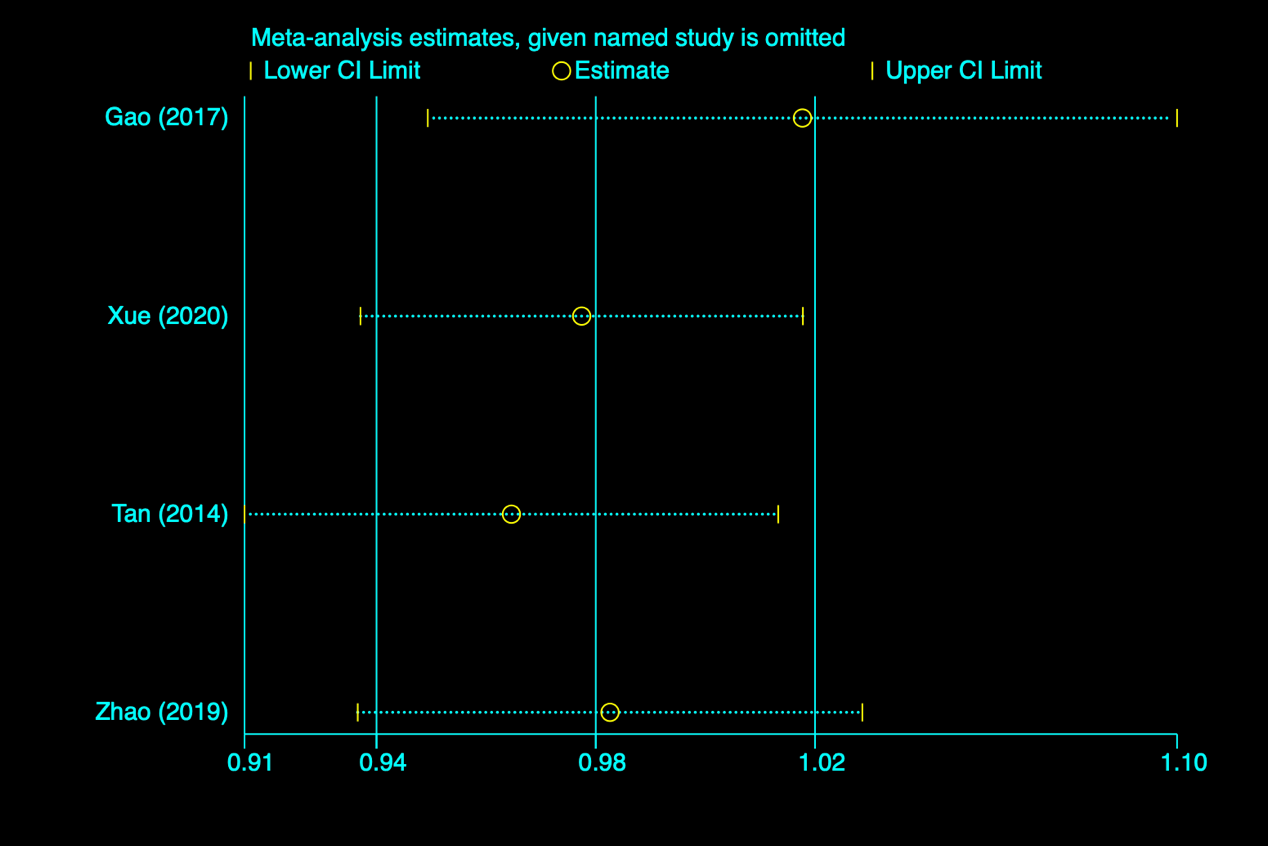


figure s2 Sensitivity analysis of treatment time


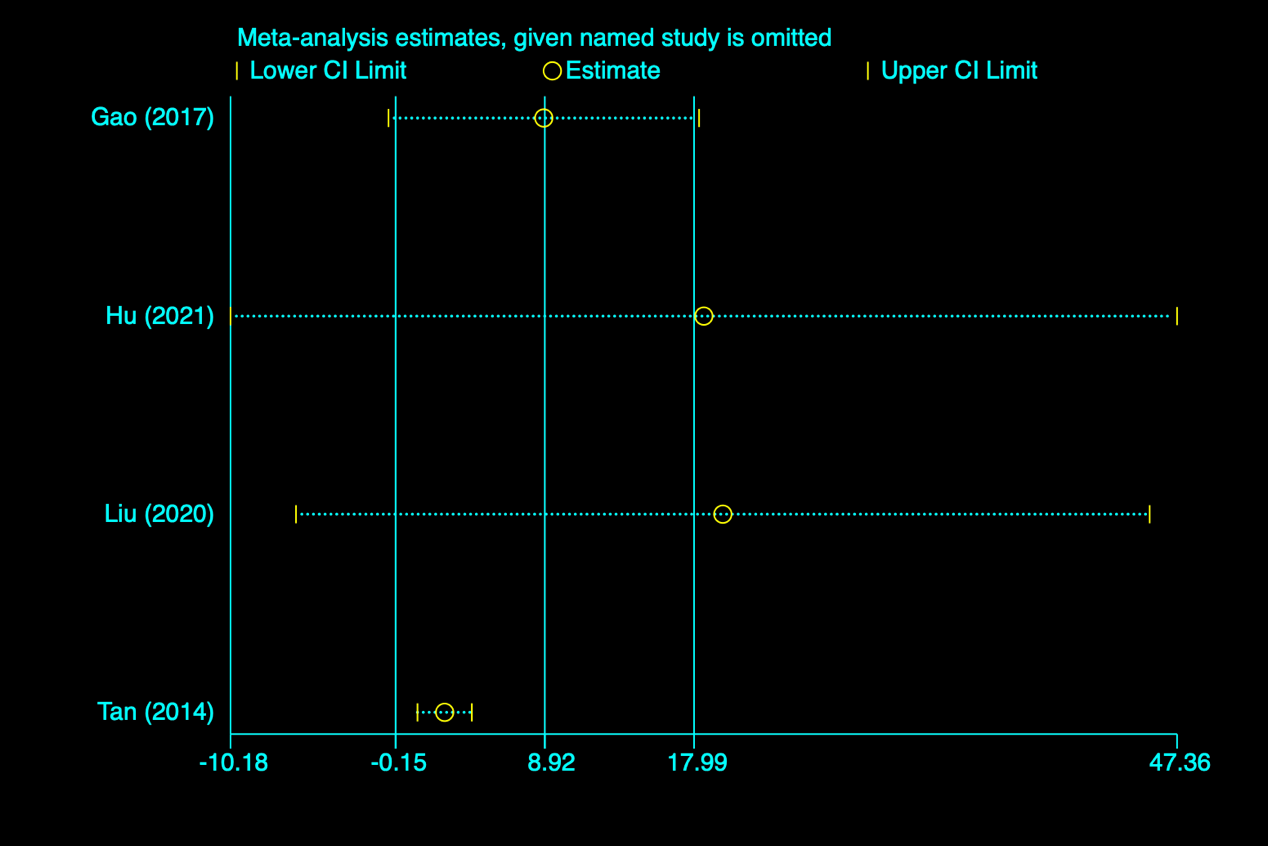


figure s3 Sensitivity analysis of surgery
